# Antibacterial Activities of *Jatropha curcas* (LINN) on Coliforms Isolated from Surface Waters in Akure, Nigeria

**Published:** 2014-03

**Authors:** E. O. Dada, F. O. Ekundayo, O. O. Makanjuola

**Affiliations:** Department of Microbiology, Federal University of Technology, PMB 704, Akure, Nigeria

**Keywords:** coliforms, Jatropha curcas, phytochemicals, surface waters

## Abstract

This study investigated the antibacterial activities of hot water, ethanol and acetone extracts of *Jatropha curcas* (LINN) leaves on coliforms isolated from surface waters using growth inhibition indices based on agar plate technique. The percentage recovery of the extracts was 19.17%, 18.10% and 18.80% for hot water, ethanol and acetone respectively. Phytochemical screening of the extracts was also determined. Qualitative phytochemical screening showed that the plant extracts contained steroids, tannins, flavonoids and cardiac glycosides, while alkaloids, phlobatannin, terpenoids and anthraquinones were absent. Only ethanolic extract did not possess saponins. Aqueous extracts of *J. curcas* compared most favourably with the standard antibiotics (gentamycin) on all the coliform bacteria except on *K. pneumoniae* and *E. coli* likely due to a measurably higher antibacterial activity compared to the organic extracts. The minimum inhibitory concentration of the aqueous extract ranged from 3.00 to 7.00 mg/L while minimum bactericidal concentration ranged from 4.00 to 10.00 mg/L. Aqueous extract of *J. curcas* could be used as antibacterial agents against diseases caused by coliforms.

## INTRODUCTION

Coliform bacteria are Gram-negative, facultatively anaerobic, nonspore-forming bacilli that ferment lactose with gas production (5). They belong to the Enterobacteriaceae family, commonly known as enteric bacteria, meaning they reside in the gastrointestinal tract and are usually shed from the body in faecal material. Coliforms appear in great quantities in the gastrointestinal tracts and faeces of people and endothermic, or warm-blooded, animals (3, 26). The concept of indicator organisms is a principal component of water quality standards and regulatory microbiology. If water quality is to be properly assessed, it is useful to determine the hosts of the indicator bacteria present in surface waters and the potential pathogens associated with them to construct a source material budget of bacterial inputs, as management strategies from human sources are likely to be different than those from animal sources (8, 20). Waterborne and water related diseases such as diarrhea, typhoid, cholera and drancunculiasis are fast becoming endemic in certain parts of Africa (31, 39).

Traditional medicine using plant extracts continues to provide health coverage for over 80% of the world’s population, especially in developing countries (27). It has been reported that Africa has as much as 300,000 medicinal plants (39). There is, therefore, an urgent need to explore and utilize these rich biodiversity through researches that could translate to direct benefit to humankind (40). Also, the search for new antibacterial drugs of natural origin is urgently needed in the light of growing cases of microbial resistance to the available synthetic antibiotics (17, 23, 38). *Jatropha curcas* L. (Euphorbiaceae) or physic nut is a large drought-resistant shrub that is used for several purposes (29). Itis widely used in traditional medicine in Africa, Asia and Latino America to cure various ailments such as skin infections, diarrhea, gonorrhea, fever and several other diseases caused by microorganisms (6, 18, 25). *Jatropha curcas* has also been used as antidote, remedy, medicine and potential source of herbal drugs in dental complaints and against constipation (4). The milky sap is used for the treatment of dermatomucosal diseases. The leaves are used to make tea to treat malaria and the sap to stop breeding (4). Kaushik *et al.* (19) reported that *J. curcas* contains an alkaloid known as jatrophine which is believed to have anti-cancerous properties. The latex can be used as a remedy for alopecia, anasorca, burns, dropsy, eczema, inflammation, paralysis and yellow fever.

Previous studies have reported that *J. curcas* exhibits antimicrobial activity (1, 2, 10, 15), however ,there is dearth of information on the antibacterial activities of *J. curcas* on coliforms isolated from surface waters in Akure, Ondo State, Nigeria. Therefore, the present investigation is aimed at *in vitro* screening of the antibacterial properties of crude extracts of *J. curcas* and to establish its effectiveness in the treatment of diseases caused by coliforms.

## MATERIALS AND METHODS

### Collection and preparation of plant materials

Fresh leaves of *Jatropha curcas* were collected from a farm settlement at Ado-Ekiti, Ekiti State, Nigeria. The leaves were identified and authenticated at the museum of the Department of Crop, Soil and Pest Management, the Federal University of Technology, Akure (FUTA), Ondo State, Nigeria. Extracts were prepared as described by Harbone (13) with slight modifications. The leaves were air dried for three weeks and pulverized using an electric blender (Marlex Electrolyne IS: 250). The solvents used for the extraction were 100% ethanol, acetone and hot water. Exactly 200 g of the powdered leaf was soaked in each solvent. Each solution was allowed to stand for 72 hours, after which it was sieved with a muslin cloth and filtered using No 1 Whatman filter paper. The filtrate was collected in a beaker and concentrated in a *vacuo* using rotary evaporator (Resona, Germany). The extracts were reconstituted in tween 20 (10% v/v) prior to use and sterilized with the aid of membrane filter (0.22 μm). The dry weights of the dried extracts were measured and reported.

$$\text{Percentage extract recovery}\frac{\text{Dry weight of extract recovered after extraction}}{\text{Initial dry weight of plant part}}\times \;100\%$$

### Fractionation of extracts

Chloroform fraction (CF) was obtained by fractionating one gram of the extract using column chromatography (CC) (Si gel column, 60 g, 250 ml burette). Fractions (100 ml) were eluted using chloroform. Main fractions were pooled together and were rechromatographed using short column (Si gel column, 30 g, 25 × 1 cm). The second fraction (C2) was further purified using thin layer chromatography (TLC) (Si gel TLC, 60 g, 250 ml burette) using chloroform-methanol (9.5: 0.5 v/v) as the solvent system; it gives a yellow colour. This was done according to Philip (30).

### Phytochemical analysis of the plant extract

The extracts were screened for phytochemicals such as alkaloids, flavonoids, tannins, saponins, steroid, alkaloids and glycosides in accordance with Trease and Evans (36).

### Bacteria strains

The coliforms used in this investigation were obtained from Microbiology Department, Federal University of Technology and were previously isolated from surface water samples in Akure, Ondo State, Nigeria and they include *Escherichia coli, Enterobacter aerogenes, Serratia marcescens, Klebsiella pneumoniae* and *Citrobacter freundii*.

### Assay for antibacterial activity of *J. curcas* extracts

Antibacterial activities of the plant extracts were determined by the agar well diffusion method as described by Esimore *et al* (12). Different concentrations of 12.5 mg/ml, 25 mg/ml, 50 mg/ml, 100 mg/ml, 200 mg/ml and 400 mg/ml of the extracts were used for the bioassay. The 12.5 mg/ml chloroform fraction of the extract was also assayed. After incubation, zones of inhibition formed in the medium were measured in millimeter (mm) diameter. Gentamycin (10 μg) was used as standard antibacterial agent for positive control.

### Determination of minimum inhibitory concentration (MIC) and minimum bactericidal concentration (MBC) of the leaf extract of *J. curcas*

The MIC of isolates was carried out using tube dilution technique as described by Doughari *et al* (9). McFarland turbidiometric standard (10^6^cfu/ml) was used to standardize the concentration of test coliforms. A tube containing 2 ml of 18 hrs nutrient broth without extract was seeded with a loopful of the test organism previously diluted to 0.5 McFarland turbidiometric standard to serve as the positive control while a tube containing 2 ml of 18 hrs nutrient broth that was not inoculated served as the negative control. After incubation for 24 hours at 37°C, the tubes were then examined for microbial growth by observing the turbidity. To determine the MBC, for each set of test tubes in the MIC determination, a loopful of broth was collected from those tubes which did not show any visible sign of growth and inoculated on sterile nutrient agar by streaking. Nutrient agar plates were streaked with the test organisms only to serve as control. The plates were then incubated at 37°C for 24 hours. After incubation, the concentration at which no visible growth was seen was recorded as the minimum bactericidal concentration.

### Statistical analysis

All experiments were carried out in triplicate. Data obtained were analyzed by one way analysis of variance (ANOVA) and means were compared by Duncan’s New Multiple Range test using SPSS 16.0 version and means were separated by least significant differences (*P*≤0.05).

## RESULTS

There was no significant difference in the percentage yield of extracts obtained although aqueous extract had the highest yield of 19.17% and the least recovery was obtained for acetone extract with 18.10% (Table 1). Qualitative phytochemical screening showed that the plant extracts contained steroids, tannins, flavonoids and cardiac glycosides, while alkaloids, phlobatannin, terpenoids and anthraquinones were absent. Only ethanolic extract did not possess saponins (Table 2).

**Table 1:** Percentage recovery of *J. curcas* leaf extracts

Solvents	Amount extracted (g) ± SD	Percentage recovery (%) ± SD

Hot Water	38.34 ± 2.11	19.17 ± 3.05
Ethanol	37.60 ± 3.08	18.80 ± 3.76
Acetone	36.20 ± 1.55	18.10 ± 1.20

Values are means ± standard deviation for three samples. Mean followed by the same letter(s) within the group are not significantly different at *p*≤0.05 using duncan’s new multiple range test. SD, Standard deviation.

**Table 2:** Qualitative analysis of phytochemicals of *J. curcas* leaf extracts

Phytochemicals	Ac	E	Aq

Alkaloids	-	-	-
Tannins	+	+	+
Saponins	+	-	+
Phlobatannins	-	-	-
Anthraquinones	-	-	-
Flavonoids	+	+	+
Steroids	+	+	+
Terpenoids	-	-	-
Cardiac Glycosides	+	+	+

Aq, aqueous; Ac, acetone; E, ethanol; +, positive; -, negative.

The ethanolic and acetone extracts of *J. curcas* leaves had no antibacterial activity except on *Kl. pneumoniae* (4.0 mm) and *S. typhi* (3.0 mm) at concentration of 400 mg/ml. The aqueous extract of *J. curcas* leaves exhibited broad spectrum activity on the test coliform bacteria at concentration of 400mg/ml. Relative to the crude extracts at concentration of 12.5 mg/ml, the chloroform fraction had higher zones of inhibition on all isolates except on *P. vulgaris, C. freundii, E. coli* and *Sh. dysenteriae* in aqueous extracts. Gentamycin (10μg), the reference antibiotic which also served as the positive control had higher zones of inhibition on only *Kl. pneumoniae* and *E. coli* as compared to the crude and purified extracts. There was significant difference between the antibacterial activities of crude and fractionated extracts (Table 3).

**Table 3:** Antimicrobial activities of *J. curcas* extracts on coliforms isolated from surface water

Extract	Conc. (mg/ml)	ZONES OF INHIBITION (mm) ± SD							
		SHI	SAL	SER	KLE	ENT	ESC	CIT	PRO

AQUEOUS	400	13.00 ± 0.01^f^	15.00 ± 0.00^f^	16.00 ± 0.00^g^	14.17 ± 0.15^f^	9.10 ± 0.10^e^	14.10 ± 0.01^e^	15.51 ± 0.01^f^	13.50 ± 0.10^f^
	200	13.00 ± 0.01^f^	15.00 ± 0.10^f^	16.00 ± 0.01^g^	14.10 ± 0.10^e^	9.10 ± 0.01^e^	14.10 ± 0.17^e^	15.51 ± 0.01^f^	13.50 ± 0.10^f^
	100	12.01 ± 0.01^e^	14.00 ± 0.00^e^	15.10 ± 0.17^f^	14.01 ± 0.12^d^	8.01 ± 0.01^d^	13.02 ± 0.01^d^	15.50 ± 0.00^e^	11.50 ± 0.10^e^
	50	12.00 ± 0.01^e^	14.00 ± 0.06^e^	15.00 ± 0.00^e^	10.00 ± 0.01^c^	8.01 ± 0.01^d^	11.00 ± 0.06^c^	12.10 ± 0.10^d^	10.01 ± 0.01^d^
	25	10.00 ± 0.01^d^	11.07 ± 0.12^d^	12.02 ± 0.01^c^	10.00 ± 0.00^c^	5.10 ± 0.10^b^	11.00 ± 0.00^c^	9.01 ± 0.01^c^	10.00 ± 0.01^d^
	12.5	7.03 ± 0.01^c^	8.00 ± 0.01^b^	11.10 ± 0.01^b^	5.10 ± 0.01^a^	5.10 ± 0.10^b^	8.10 ± 0.10^b^	9.00 ± 0.01^c^	6.00 ± 0.01^c^
Chloroform fraction	12.5	4.60 ± 0.10^b^	9.00 ± 0.25^c^	14.50 ± 0.50^d^	9.00 ± 0.25^b^	5.80 ± 0.10^c^	3.60 ± 0.15^a^	5.10 ± 0.05^b^	4.50 ± 0.05^b^
Gentamycin	10 (μg)	0.00^a^	2.33 ± 1.53^a^	4.33 ± 1.52 ^a^	18.67 ± 3.06^g^	4.00 ± 1.00^a^	24.67 ± 2.51^f^	3.00 ± 0.25^a^	0.00a
ACETON^e^	400	0.0^a^	0.0^a^	0.0^a^	4.10 ± 0.01^d^	0.0^a^	0.0^a^	0.0^a^	0.0^a^
	200	0.0^a^	0.0^a^	0.0^a^	4.06 ± 0.10^d^	0.0^a^	0.0^a^	0.0^a^	0.0^a^
	100	0.0^a^	0.0^a^	0.0^a^	2.16 ± 0.04^c^	0.0^a^	0.0^a^	0.0^a^	0.0^a^
	50	0.0^a^	0.0^a^	0.0^a^	1.02 ± 0.02^b^	0.0^a^	0.0^a^	0.0^a^	0.0^a^
	25	0.0^a^	0.0^a^	0.0^a^	0.0^a^	0.0^a^	0.0^a^	0.0^a^	0.0^a^
	12.5	0.0^a^	0.0^a^	0.0^a^	0.0^a^	0.0^a^	0.0^a^	0.0^a^	0.0^a^
Chloroform fraction	12.5	4.60 ± 0.10^b^	9.00 ± 0.25^c^	14.50 ± 0.50^c^	9.00 ± 0.25^e^	5.80 ± 0.10^c^	3.60 ± 0.15^b^	5.10 ± 0.05^c^	4.50 ± 0.05^b^
Gentamycin	10 (μg)	0.00^a^	2.33 ± 1.53^b^	4.33 ± 1.52 ^b^	18.67 ± 3.06^f^	4.00 ± 1.00^b^	24.67 ± 2.51^c^	3.00 ± 0.25^b^	0.00a
ETHANOL	400	0.0^a^	3.21 ± 0.02^d^	0.0^a^	4.26 ± 0.02^d^	0.0^a^	0.0^a^	0.0^a^	0.0^a^
	200	0.0^a^	1.10 ± 0.01^b^	0.0^a^	4.18 ± 0.01^c^	0.0^a^	0.0^a^	0.0^a^	0.0^a^
	100	0.0^a^	0.0^a^	0.0^a^	1.02 ± 0.04^b^	0.0^a^	0.0^a^	0.0^a^	0.0^a^
	50	0.0^a^	0.0^a^	0.0^a^	0.0^a^	0.0^a^	0.0^a^	0.0^a^	0.0^a^
	25	0.0^a^	0.0^a^	0.0^a^	0.0^a^	0.0^a^	0.0^a^	0.0^a^	0.0^a^
	12.5	0.0^a^	0.0^a^	0.0^a^	0.0^a^	0.0^a^	0.0^a^	0.0^a^	0.0^a^
Chloroform fraction	12.5	4.60 ± 0.10^b^	9.00 ± 0.25^e^	14.50 ± 0.50^c^	9.00 ± 0.25^e^	5.80 ± 0.10^c^	3.60 ± 0.15^b^	5.10 ± 0.05^c^	4.50 ± 0.05^b^
Gentamycin	10 (μg)	0.00^a^	2.33 ± 1.53^c^	4.33 ± 1.52^b^	18.67 ± 3.06^f^	4.00 ± 1.00^b^	24.67 ± 2.51^c^	3.00 ± 0.25^b^	0.00^a^

Mean followed by the same letter(s) within the group are not significantly different at *p*≤0.05. Legend: SHI, *Shigella dysenteriae*; SAL, *Salmonella typhi*; SER, *Serratia marcescens*; KLE, *Klebsiella pneumoniae*; ENT, *Enterobacter aerogenes*; ESC, *Escherichia coli*; CIT, *Citrobacter freundii*; PRO, *Proteus vulgaris*; SD, Standard deviation.

The minimum inhibitory concentrations ranged from 3.0 mg/ml to 160.0 mg/ml while minimum bactericidal concentrations ranged from 4.0 mg/ml to 180.0 mg/ml as shown in Table 4.

**Table 4:** Minimum inhibitory concentration (mg/ml) and minimum bactericidal concentration (mg/ml) of *Jatropha curcas* leaf extracts

Organisms	MIC			MBC		
	Hot Water	Acetone	Ethanol	Hot Water	Acetone	Ethanol

SHI	4.00	ND	ND	5.00	ND	ND
SAL	4.00	ND	160.00	4.00	ND	180.00
SER	4.00	ND	ND	4.00	ND	ND
KLE	3.00	45.00	90.00	6.00	45.00	90.00
ENT	6.00	ND	ND	10.00	ND	ND
ESC	4.00	ND	ND	5.00	ND	ND
CIT	5.00	ND	ND	5.00	ND	ND
PRO	7.00	ND	ND	8.00	ND	ND

SHI, *Shigella dysenteriae*; SAL, *Salmonella typhi*; SER, *Serratia marcescens*; KLE, *Klebsiella pneumoniae*; ENT, *Enterobacter aerogene*; ESC, *Escherichia coli*; CIT, *Citrobacter freundii*; PRO, *Proteus vulgaris*; ND, Not Determined; MIC, Minimum inhibitory concentration; MBC, Minimum bactericidal concentration.

## DISCUSSION

Difference in percentage recovery and phytochemical analysis observed in studied plant extracts may have resulted from various solvents as reported by Kordali *et al*. (22). Hot water was most effective in the extraction of *J. curcas* leaf; this may indicate the presence of heat-labile and few polar compounds in *J. curcas* leaf. This observation is supported by Cowan (7) who reported that the most active components are generally insoluble, hence it is expected that low polarity organic solvents would yield more percentage recovery. Therefore, Takazawa *et al.* (35) suggested that there is a need to employ broad range of solvents in the extraction of phytochemicals from medicinal plants.

The qualitative phytochemical analysis indicated the presence of active constituents in plant leaf extract. This finding was in agreement with previous works of El Diwani *et al.* (11) who reported the presence of saponins in *Jatropha curcas* leaf. The absence of alkaloids in *Jatropha curcas* leaf extracts had also been reported by Kubmarawa* et al.* (24) although Igbinosa *et al*. (15) and Akinpelu *et al*. (2) observed the presence of alkaloids in *J. curcas* stem bark and leaves extracts respectively. These compounds have been associated with medicinal uses for centuries and were reported as the most efficient, therapeutically significant plant substance (27, 28) and exert antibacterial activity through different mechanisms (32, 33).

The qualitative difference of phytochemical analysis observed in plant extracts may be attributed to different solvents used for extraction. This observation is in line with the findings of Srinivasan *et al.* (34) and Kordali *et al*. (22) that reported different solvents have different spectrum of solubility for the phytoconstituents. In addition, difference in phytochemical analysis could have been as a result of varying habitats for plant growth. This observation is in agreement with Farooq *et al.* (13) who reported that plants occur in varying habitats, and explained the great magnitude of variation in the concentration and composition of phytochemical ingredients in the different parts of these plants.

Only the aqueous extracts of *J. curcas* compared favourably with the standard antibiotics (gentamycin) on all the coliform bacteria except *K. pneumoniae* and *E. coli*. The results also showed that the aqueous extracts of *J. curcas* leaf had higher antibacterial activity compared to its organic extracts. This could be as a result of its relatively high percentage extract recovery.This is in agreement with the findings of Srinivasan *et al*. (34) who reported that different solvents have different extraction capacities and different spectrum of solubility for the phyto-constituents which are known to be biologically active. The inhibitory activity of plant extract is also largely dependent on the concentration, parts of the plant used and the microbes tested (17). Although it has been stated that aqueous extracts of plant generally showed little or no antibacterial activities (21, 29), the results of the present investigation proved otherwise.

## CONCLUSION

The present investigations has shown that aqueous extract of *J. curcas* leaves showed potent antibacterial activities on coliforms than methanol and acetone extracts with the MIC values ranging from 3.00 to 7.00 mg/L while MBC ranged from 4.00 to 10.00 mg/L. Hence, aqueous extract of *J. curcas* could be used as antibacterial agents against diseases caused by the isolated coliforms from surface waters although toxicological study is recommended to be carried out administration to ensure human safety.
